# New Cases and Mutations in *SEC23B* Gene Causing Congenital Dyserythropoietic Anemia Type II

**DOI:** 10.3390/ijms24129935

**Published:** 2023-06-09

**Authors:** Melina Mara Musri, Veronica Venturi, Xènia Ferrer-Cortès, Lídia Romero-Cortadellas, Gonzalo Hernández, Pilar Leoz, María Pilar Ricard Andrés, Marta Morado, María del Carmen Fernández Valle, David Beneitez Pastor, Ana Ortuño Cabrero, Maite Moreno Gamiz, Leonor Senent Peris, Amanda Isabel Perez-Valencia, Santiago Pérez-Montero, Cristian Tornador, Mayka Sánchez

**Affiliations:** 1 BloodGenetics S.L. Diagnostics in Inherited Blood Diseases, 08950 Esplugues de Llobregat, Spainsperez@bloodgenetics.com (S.P.-M.); ctornador@bloodgenetics.com (C.T.); 2 Department of Basic Sciences, Iron Metabolism: Regulation and Diseases Group, Universitat Internacional de Catalunya, 08195 Sant Cugat del Vallès, Spain; 3 Red Blood Cell Disorders Unit, Department of Hematology, Hospital de la Santa Creu i Sant Pau, 08025 Barcelona, Spain; 4 Hematology and Hemotherapy, Hospital Universitario Fundación Alcorcón, Avda Budapest, 28922 Alcorcon, Spain; 5 Department of Hematology, University Hospital La Paz, 28046 Madrid, Spain; 6 Hospital Universitario Puerta del Mar, 11009 Cádiz, Spain; 7 Red Blood Cell Disorders Unit, Hematology Department, Hospital Universitari Vall d’Hebron, VHIO, VHIR, 08035 Barcelona, Spain; 8 Hospital Universitario Cruces, 48903 Barakaldo, Spain; 9 Laboratory of Cytomorphology, Unity of Hematologic Diagnostic, Hospital Universitari i Politècnic La Fe, 46026 Valencia, Spain; 10 Department of Hematology, Hospital Clínic de Barcelona, 08036 Barcelona, Spain

**Keywords:** congenital dyserythropoietic anemia, CDA type II, SEC23B, hereditary anemias, ineffective erythropoiesis, rare blood disease, mutations, variants

## Abstract

Congenital dyserythropoietic anemia type II (CDA II) is an inherited autosomal recessive blood disorder which belongs to the wide group of ineffective erythropoiesis conditions. It is characterized by mild to severe normocytic anemia, jaundice, and splenomegaly owing to the hemolytic component. This often leads to liver iron overload and gallstones. CDA II is caused by biallelic mutations in the *SEC23B* gene. In this study, we report 9 new CDA II cases and identify 16 pathogenic variants, 6 of which are novel. The newly reported variants in SEC23B include three missenses (p.Thr445Arg, p.Tyr579Cys, and p.Arg701His), one frameshift (p.Asp693GlyfsTer2), and two splicing variants (c.1512-2A>G, and the complex intronic variant c.1512-3delinsTT linked to c.1512-16_1512-7delACTCTGGAAT in the same allele). Computational analyses of the missense variants indicated a loss of key residue interactions within the beta sheet and the helical and gelsolin domains, respectively. Analysis of SEC23B protein levels done in patient-derived lymphoblastoid cell lines (LCLs) showed a significant decrease in SEC23B protein expression, in the absence of SEC23A compensation. Reduced *SEC23B* mRNA expression was only detected in two probands carrying nonsense and frameshift variants; the remaining patients showed either higher gene expression levels or no expression changes at all. The skipping of exons 13 and 14 in the newly reported complex variant c.1512-3delinsTT/c.1512-16_1512-7delACTCTGGAAT results in a shorter protein isoform, as assessed by RT-PCR followed by Sanger sequencing. In this work, we summarize a comprehensive spectrum of *SEC23B* variants, describe nine new CDA II cases accounting for six previously unreported variants, and discuss innovative therapeutic approaches for CDA II.

## 1. Introduction

Congenital dyserythropoietic anemias (CDAs) are a group of rare hereditary disorders characterized by ineffective erythropoiesis, different morphologic changes in erythroblasts, hemolytic anemia, and hemochromatosis. The CDAs include CDA type I to type IV and X-linked thrombocytopenia with dyserythropoietic anemia (XLTDA), all caused by mutations in different genes [1,2]. Among them, the CDA type II (CDA II, OMIM 224100) is the most common, inherited in an autosomal recessive fashion. CDA II patients exhibit a variable degree of anemia ranging from very severe, requiring red cell transfusions in utero [3,4], to moderate or mild; affected individuals with normal hemoglobin levels have also been reported [5]. Other clinical manifestations include jaundice, splenomegaly, and iron overload. Some patients may present additional complications such as leg ulcers [3], aplastic crisis [6], and bulky extramedullary erythropoiesis [7]. The bone marrow of CDA II patients is hypercellular, and usually presents more than 10% of mature bi- or multi-nucleated erythroblasts, with nuclei of equal size and DNA content [8]. Under electron microscope, late erythroblasts show an additional membrane consisting of residual endoplasmic reticulum beneath the cytoplasmic membrane [9,10]. Hypoglycosylation of erythrocyte membrane proteins [11,12] is a biochemical hallmark of CDAII: band 3 (anion exchanger 1) and band 4.5 (glucose transporter 1) are detected by immunoblotting as sharper and faster-migrating [13,14], a feature exploited as a diagnostic discriminant for CDA II detection [15].

Genome linkage analysis mapped the CDA II locus to a 5 cM region on chromosome 20q11.2 [16]; mutations in the *SEC23B* gene were later discovered as the genetic basis of this disease [17,18]. SEC23B and its paralog protein SEC23A are core components of the coat protein complex II (COPII), a vesiculation machinery that mediates anterograde transport of correctly folded cargo from the endoplasmic reticulum to the Golgi [18,19]. The *SEC23B* gene spans ~54 kb on human chromosome 20 and comprises 20 exon regions encoding 767 residues arranged in five functional domains: zinc finger, trunk, beta sheet, helical, and gelsolin domain. As characterized in *S. cerevisiae*, each Sec23bp domain interacts with other coat subunits: the trunk domain connects with Sec24p, Sar1p, and Sec31p; the gelsolin domain makes contact with Sar1p and Sec31p. Moreover, Sec31p interacts with the zinc finger, the beta sheet, and the helical domains of Sec23bp [20].

Clinically, CDA II patients present with ineffective erythropoiesis that leads to downregulation of the hepcidin hormone; this increases iron absorption and can drive systemic iron overload, a main complication in CDA II patients [15]. At the molecular level, SEC23B loss-of-function impairs the glycosylation of membrane proteins involved in the activation of bone morphogenic proteins (BMP)/SMADs signaling [21]. This alteration triggers hepcidin repression in both patients and liver cell lines, pointing to a direct role of SEC23B in hepatic iron homeostasis [21].

CDA II conventional therapy consists of red cell transfusions, iron chelation, and splenectomy. According to recent splenectomy guidelines, other than hereditary spherocytosis for which there is evidence of effectiveness, the efficacy of splenectomy in treating anemias has yet to be clarified; thrombotic complications and short- and long-term post-splenectomy infections are also of concern [22]. As for CDAs, no recommendation supporting the use of splenectomy in CDA I management could be made due to inconsistent case series results [23]. Risks for asplenic CDAII patients are weighed up by a moderate increase in their hemoglobin levels and no documented cases of thrombosis associated with splenectomy. Such surgical procedures should, however, be reserved for severely anemic patients and/or cases of symptomatic splenomegaly [22]. Allogenic hematopoietic stem cell transplant (HSCT) was successfully utilized as a curative treatment in CDA II patients [24,25]. Innovative therapies such as autologous HSCT of genetically corrected cells might represent an effective treatment for CDA II.

To date, 110 variants in the *SEC23B* gene have been described to cause CDA II (summarized in Supplementary Table S1). In this work, we describe nine CDA II patients from nine unrelated families accounting for six novel variants in the *SEC23B* gene. We address some of the biological consequences of these newly described variants by computational modeling and analysis of SEC23B mRNA and protein levels in immortalized patient-derived cells, shedding light on the mutational spectrum of CDA II.

## 2. Results

### 2.1. Review of the SEC23B Mutational Spectrum

**Figure 1:**
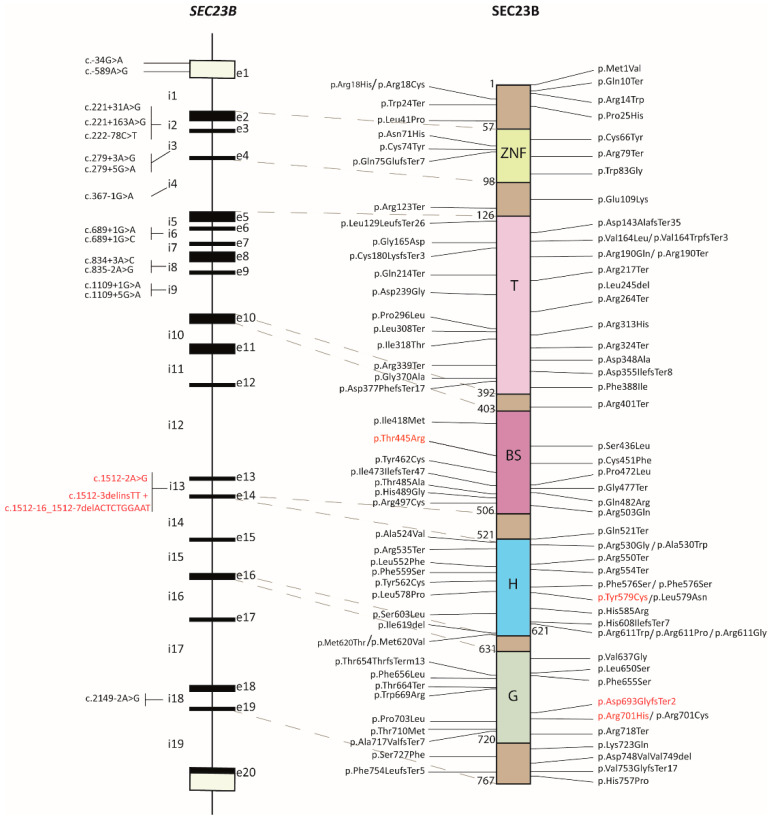
Mutational spectrum map of SEC23B. Localization of the reported pathogenic mutations in the *SEC23B* gene (**left**) and SEC23B protein (**right**). The new variants reported in this work are highlighted in red. e, exon; ivs, intervening sequence; ZNF, zinc finger domain; T, trunk domain; BS, beta sheet domain; H, helix domain; G, gelsolin domain.

We did a thorough literature and database search to collect and revise the mutational spectrum of *SEC23B* pathogenic variants associated with CDA II (Supplementary Table S1). The mutations are scattered across the length of the SEC23B protein, with no evident clustering (Figure 1). As previously reported [26], the most frequent mutations described in *SEC23B* are nucleotide missense and nonsense mutations (around 63% and 15%, respectively), whereas frameshift and indels account for about 13% and 4%. To this mutational spectrum, we add six previously undescribed variants (Figure 1, highlighted in red) of which three are missense, one is frameshift, and two are splicing variants.

### 2.2. New CDA II Cases

Here, we describe nine patients carrying variants in the *SEC23B* gene. The patients’ clinical features are summarized in Table 1. The variants described below refer to NM_032986.4 for the *SEC23B* gene and NP_116781.1 for the SEC23B protein.

#### 2.2.1. Family 1. Proband II.1

A 15-year-old female patient reported a diagnosis of CDA II done elsewhere at the hematological service of the Hospital Universitari Vall d’Hebron, Spain. Her hemoglobin level was 9.6 g/dL and her MCV level was 93.2 fL. The patient was splenectomized at the age of 7 and cholecystectomized at the age of 8. Peripheral blood analysis displayed morphological alterations secondary to splenectomy (acanthocytes, spherocytes, Howell–Jolly bodies) and irregular hemoglobin distribution. There is no relevant family history; the non-consanguineous parents did not show any hematological abnormalities.

**Figure 2:**
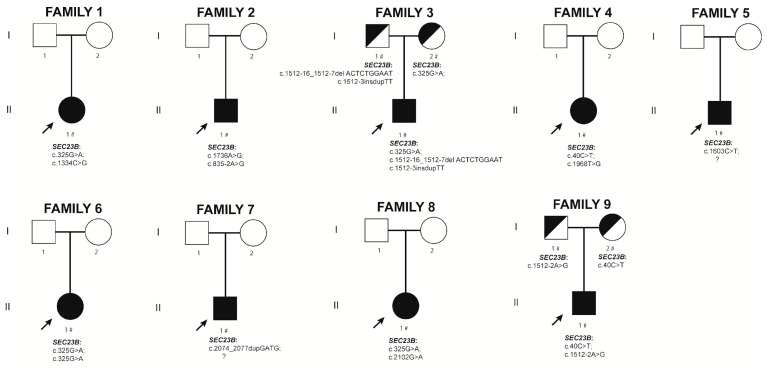
Pedigrees and variants for new CDA II cases. The probands are indicated with an arrow. Black symbols denote affected individuals and half-filled black symbols unaffected carriers. Individuals studied at the molecular level are indicated with the # symbol.

Next-generation sequencing (NGS) genetic analysis identified the previously reported missense mutation c.325G>A (p.Glu109Lys) and the novel variant c.1334C>G (p.Thr445Arg) in compound heterozygosity in the *SEC23B* gene (Figure 2). This novel variant corresponds to the rsID number rs121918221 and, according to ClinVar, it has a global minor allele frequency (GMAF) of 0.00020 and a Genome Aggregation Database (gnomAD) Genome ƒ = 0.000243. The American College of Medical Genetics and Genomics (ACMG) classification for variant c.1334C>G (p.Thr445Arg) lists it as a variant of unknown significance (VUS). VarSome Clinical platform [27] assigns to this variant a predicted damaging score using 13 pieces of software out of 20.

#### 2.2.2. Family 2. Proband II.1

A 12-year-old male patient was referred to the hematological department of the Hospital Universitario Puerta del Mar, Spain, in July 2010 with clinical symptoms suspicious of CDA. His hemoglobin level was 8.5 g/dL and his MCV level was 85 fL. Bone marrow aspirate pointed to dyserythropoietic disorders. His mother received two blood transfusions eight weeks before giving birth. Alpha thalassemia, Fanconi anemia, Diamond–Blackfan anemia, and Pearson syndrome were discarded. The patient underwent a splenectomy at the age of 9. Peripheral blood analysis showed neutrophil hypersegmentation, microcytosis, and platelet anisocytosis. Bone marrow displayed dyserythropoietic features. There is no relevant family history; the non-consanguineous parents did not show any hematological abnormalities. The patient received multiple red blood cell transfusions.

NGS genetic analysis identified the novel variant c.1736A>G (p.Tyr579Cys) and the splicing mutation c.835-2A>G in a compound heterozygosity state in the *SEC23B* gene (Figure 2). The splicing mutation c.835-2A>G has been previously described by Bianchi et al. [28] and has a gnomAD Gnomes ƒ = 0.0000788. The ACMG classification for variant c.1736A>G (p.Tyr579Cys) lists it as a VUS. VarSome Clinical platform indicates that this variant has a predicted damaging score using 14 software out of 20.

#### 2.2.3. Family 3. Proband II.1

A 32-year-old male patient was admitted to the hematological department of the University Hospital La Paz, Spain, in 2012 with anemia and splenomegaly (splenic length 17 cm) detected in the context of an acute Giardia infestation. He presented with mild chronic anemia and hyperbilirubinemia, with no previous diagnosis; his hemoglobin and MCV levels were 12.7 g/dL and 91 fL, respectively. Peripheral blood smear test exhibited anisopoikilocytosis with echinocytes, ovalocytosis, stomatocytosis, teardrop cells, and schistocytes. Bone marrow aspirate showed erythroid hyperplasia with >10% of dyserythropoiesis, abundant binucleated elements, karyorrhexis, and an increased number of histiocytes. Under the electron microscope, the mature erythroblasts showed a discontinuous double membrane as a result of vesicles loaded with proteins from the endoplasmic reticulum just beneath the plasma membrane [29]. The patient is not splenectomized. There is no relevant family history and the non-consanguineous parents did not show any hematological abnormalities. The patient was treated with Deferasirox from 2012 to 2015 due to non-transfusional iron overload; he is currently asymptomatic.

NGS genetic analysis showed heterozygosis for the previously described *SEC23B* mutation c.325G>A (p.Glu109Lys), inherited from the mother and a new complex allele variant (including c.1512-3delinsTT) linked to the intronic c.1512-16_1512-7delACTCTGGAAT deletion inherited from the father (Figure 2). The variant c.1512-3delinsTT is predicted to disrupt normal splicing according to the MutationTaster2 software [30]. The variant c.1512-16_1512-7delACTCTGGAAT corresponds to the rsID number rs2060301818. The ACMG classifies the variant c.1512-16_1512-7delACTCTGGAAT as a VUS.

#### 2.2.4. Family 4. Proband II.1

A 25-year-old female patient was referred to the hematological department of the Hospital de la Santa Creu i Sant Pau, Spain, with splenomegaly, anemia, and suspicion of congenital anemia. Her hemoglobin level was 8.2 g/dL and her MCV level was 90 fL; she mentioned suffering from anemia and jaundice at the age of 12. Bone marrow light microscope revealed erythroid hyperplasia with morphological abnormalities. Membrane protein analysis by electrophoresis showed a dense band 3 with abnormal mobility, suggestive of CDA type II. The patient underwent splenectomy and cholecystectomy at the age of 42. Magnetic resonance imaging (MRI) for quantification of liver iron done three years later displayed a liver iron concentration of 3.91 mg/g. This value indicates mild iron overload in the hepatic parenchyma, which was normalized four years later. The patient was never treated with iron chelation therapy. There is no relevant family history and the non-consanguineous parents did not show any hematological abnormalities.

NGS genetic analysis identified the missense mutations c.40C>T (p.Arg14Trp) and c.1968T>G (p.Phe656Leu) in compound heterozygosity in the *SEC23B* gene (Figure 2). Both mutations were previously described by both Schwarz’s and Iolascon’s groups as pathogenic causing CDA II [18,31]. The pArg14Trp mutation corresponds to the rsID number rs121918222 and has a gnomAD Genomes ƒ = 0.00017; the p.Phe656Leu mutation has a gnomAD Genomes ƒ = 0.0000197.

#### 2.2.5. Family 5. Proband II.1

A 44-year-old male patient was referred to the hematology unit of the Hospital Universitario Fundación Alcorcón, Spain, because of a clinical CDA II diagnosis received at the age of 21. Clinical monitoring done in 2012 showed posterior mediastinal masses with a histopathologic diagnosis of extramedullary hematopoiesis and splenomegaly (splenic length 23 cm). He suffered from iron overload with affected hepatic biochemical functions, underwent cholecystectomy, and remained untransfused. Hemochromatosis-related genotype—i.e., *HFE* genes—was excluded. The family history is not available because the patient preferred not to disclose it. Baseline hemoglobin level was 12–13 g/dL and MCV level was around 90 fL; the patient also showed hyperbilirubinemia (bilirubin 5–7 mg/dL). Neither bone marrow aspirate nor bone biopsy was performed because of a previously established diagnosis. Peripheral blood smear test showed red blood cells with anisopoikilocytosis, remarkable spherocytosis, and orthochromatic binucleated erythroblasts. In 2013, due to high ferritin levels (1332 mg/mL), the patient was put on a daily Deferasirox treatment (10–15 mg/Kg). One year later, his ferritin had decreased to 485 mg/mL and his iron liver concentration was estimated by MRI in 5.69 mg/g of liver tissue. In 2017, the patient achieved normal liver biochemical parameters and his iron liver concentration was quantified in 2 mg/g by MRI. Hyperbilirubinemia remained unresolved. Currently, the patient presents normal ferritin levels and liver iron concentration.

NGS genetic analysis identified the nonsense mutation c.1603C>T (p.Arg535Ter) in heterozygosity in the *SEC23B* gene (Figure 2). This mutation has previously been described by Russo and colleagues [20] as pathogenic, causing CDA II. It corresponds to the rsID number rs201921350 and has a gnomAD Genome ƒ = 0.0000197. After a thorough revision of the NGS panel sequences, we were unable to identify a second, causative mutation in this patient. We cannot exclude deep intronic pathogenic variants or mutations in the promoter region.

#### 2.2.6. Family 6. Proband II.1

An 18-year-old female patient was referred to the hematological department of La Paz University Hospital, Spain, for diagnosis and treatment of moderate anemia present since childhood. Her hemoglobin level was 9.8 g/dL and her MCV level was 92 fL. Peripheral blood exhibited anisopoikilocytosis, anisochromia, scanty spherocytes, schistocytes, and irregularly contracted cells. Bone marrow aspirate showed erythroid hyperplasia with pyknosis, karyorrhexis, and 25% of binucleated mature erythroblasts. Other features included neonatal jaundice and treatment with oral iron for unexplained anemia at the age of 11. The patient was transfused with two packed red blood cells at the age of 19 and with four packed red blood cells at the age of 20. In the same year, she underwent a splenectomy, followed by a cholecystectomy two years later. There is no relevant family history and the non-consanguineous parents did not show any hematological abnormalities.

NGS genetic analysis identified the previously reported missense mutation c.325G>A (p.Glu109Lys) in a homozygous state in the *SEC23B* gene (Figure 2).

#### 2.2.7. Family 7. Proband II.1

A 10-year-old male patient was referred to the hematological department of the Hospital Universitario Cruces Barakaldo, Spain, with chronic anemia. His hemoglobin level was 8.3 g/dL and his MCV level was 91 fL at the time of diagnosis. Bone marrow aspirate showed erythroid line hyperplasia (83% of bone marrow cellularity) with 20–30% of multinucleated elements, pyknosis, and karyorrhexis indicating dyserythropoiesis. Isolated pseudo-Gaucher type cells were observed. Other relevant clinical features are anemia since he was 5 years old and increased iron tissue deposits with hepatosplenomegaly. During childhood, the patient’s hemoglobin level was 7.5–9 g/dL and his platelet count was 50–70 × 10^9^/L. He presented with severe liver and cardiac iron overload and underwent a splenectomy at the age of 40. There is no relevant family history and the non-consanguineous parents did not show any hematological abnormalities.

NGS genetic analysis confirmed heterozygosity for the novel frameshift variant c.2074_2077dupGATG (p.Asp693GlyfsTer2). This variant has a gnomAD ƒ = 0.0000131 and corresponds to the rsID number rs1468581868. The ACMG classifies the variant c.2074_2077dupGATG as pathogenic, with no pathogenicity scores included. After an exhaustive analysis of the NGS panel sequences, we were unable to identify a second causative variant; however, deep intronic pathogenic variants or promoter variants cannot be excluded.

#### 2.2.8. Family 8. Proband II.1

A 24-year-old patient, diagnosed with CDA II at 8 years of age, was admitted to the pediatric department of the Hospital Universitari i Politècnic La Fe, Spain, in the outpatient clinic. The patient suffered from cholelithiasis (gallstones of 8 mm in size) at the age of 9 and underwent a cholecystectomy a year later. Bone marrow analysis performed at the age of 8 highlighted erythroid hyperplasia with a predominance of binucleate polychromatophilic forms in more than 10% of them, compatible with CDA type II. The erythroblasts were negative for periodic acid-Schiff (PAS) stain. On Perls’ stain, no pathological or ringed sideroblasts were observed. There is no relevant family history and the non-consanguineous parents did not show any hematological abnormalities. The patient is not splenectomized. The patient is transfused every three months and presents with an anemic syndrome with moderate efforts.

NGS genetic analysis identified the previously reported pathogenic missense mutation c.325G>A (p.Glu109Lys) and the new missense variant c.2102G>A (p.Arg701His) in compound heterozygous state in the *SEC23B* gene (Figure 2). Variant p.Arg701His corresponds to the rsID number rs772417195 and has a gnomAD Exome ƒ = 0.00000795. The ACMG classification for variant c.2102G>A lists it as likely pathogenic. VarSome Clinical indicates that this variant has a predicted damaging score using 16 pieces of software out of 19.

#### 2.2.9. Family 9. Proband II.1

A 9-year-old male, previously diagnosed with pulmonary valve stenosis, was referred to the hematological department of the Sant Joan de Deu pediatric hospital in Barcelona, Spain, for the study of a Coombs-negative hemolytic anemia. The initial hemoglobin level was 10.5 g/dL with an MCV of 90 fL. Peripheral blood analysis revealed anisopoikilocytosis, spherocytes and the presence of dacrocytes. Erythrocyte enzymopathy tests showed normal values of G6PD and pyruvate kinase; hemoglobin analysis showed age-adjusted normal distribution of HbF and HbA2. A bone marrow aspirate led to a diagnosis of congenital dyserythropoietic anemia. At the age of 12, the patient suffered a parvovirus infection requiring one packed red blood cell transfusion. At the age of 17, a hepatic iron overload screening was begun and performed yearly, with hepatic MRI showing hepatic iron load values between 95–150 µmol/g (normal value < 55µmol/g)) and hepatosplenomegaly. Therefore, iron chelation therapy was started in 2017 and is ongoing.

NGS genetic analysis identified the previously described missense mutation c.40C>T (p.Arg14Trp) and the unreported splicing variant c.1512-2A>G in compound heterozygosity in the *SEC23B* gene. The ACMG classified the NM_001172745.1: c.1512-2A>G variant as likely pathogenic and VarSome platform lists it as damaging using three pieces of software out of six. The MutationTaster2 software predicts this variant to cause an alteration within the splice site, likely affecting normal splicing.

### 2.3. Computational Model of p.Thr445Arg, p.Tyr579Cys, and p.Arg701His Variants

**Figure 3:**
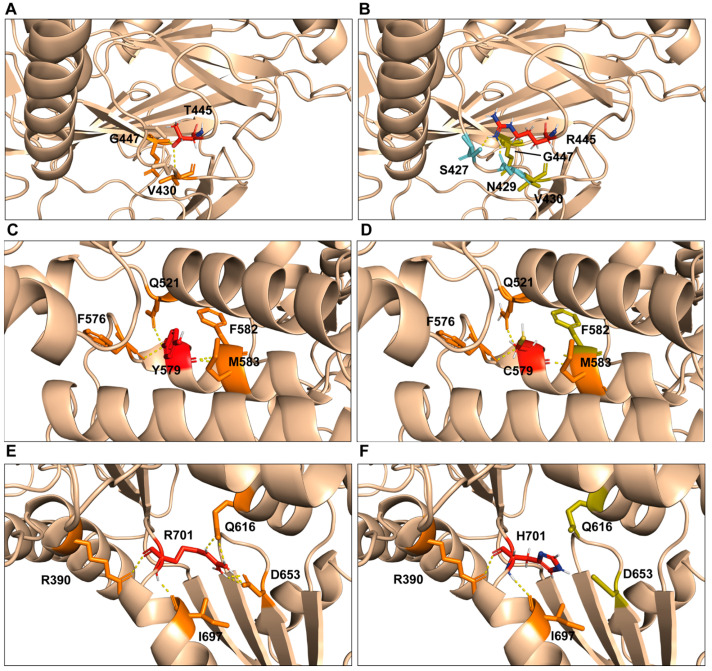
Computational model of the p.Thr445Arg, p.Tyr579Cys, and p.Arg701His variants. Native conformations are shown in panels (**A**,**C**,**E**) respectively. The three nonsynonymous substitutions induce protein structure alterations, which range from pronounced in the p.Thr445Arg (**B**) and p.Arg701His (**F**) variants to mild in p.Tyr579Cys (**D**); all changes potentially impact protein functionality.

The computational model of the human SEC23B protein shows that the Thr445 residue is located on a beta sheet domain loop and its side chain interacts with the ones of the Val430 and Gly447 residues in a nearby loop of the same domain (Figure 3A). The modeling of the p.Thr445Arg variant suggests an important structural change surrounding these structures. The longer side chain of the arginine perturbs its nearby interactions: the amino acid loses contact with the Val430 and Gly447 residues while establishing new H-bonds with Ser429 and Asn429 (Figure 3B).

The Tyr579 residue is located on the helical domain where it faces the internal region delimited by three alpha helices; there, it establishes several H-bonds via its main chain with the Gln521, Phe576, Phe582, and Met583 residues (Figure 3C). The tyrosine-to-cysteine change weakly affects these interactions, only disrupting the one with the Phe582 residue (Figure 3D). Tyrosine is a hydrophobic amino acid, whereas cysteine is viewed as a relatively polar residue (although its polarity may vary, modulated by the environment in which the residue occurs); this switch in side chain polarity might affect the cluster of the three alpha helices where this residue lays.

The Arg701 residue is located on the gelsolin domain and establishes several H-bonds with Gly616 and Asp653 in an adjacent loop, suggesting a structural role for the Arg701 residue (Figure 3E). The arginine-to-histidine change observed in this patient abolishes such interactions, presumably causing a structural change in the protein (Figure 3F).

### 2.4. Analysis of SEC23B mRNA and Protein Levels in Patient-Derived LCLs

**Figure 4:**
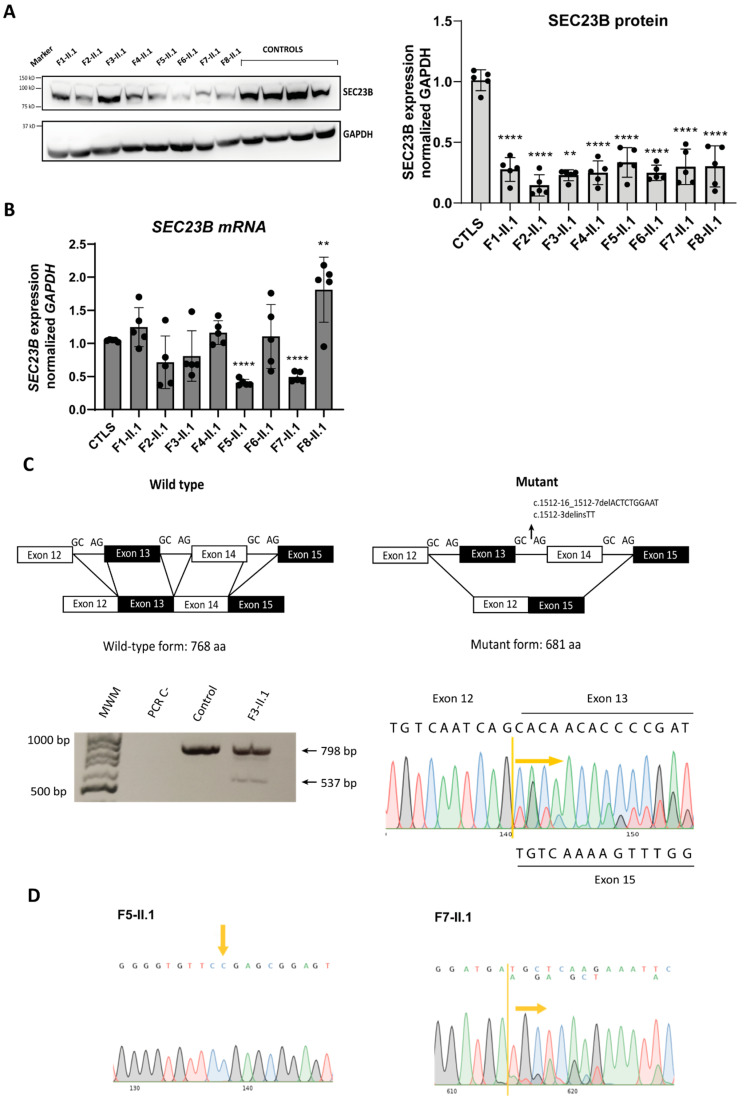
Expression of *SEC23B* mRNA and SEC23B protein. (**A**) Representative Western blot of SEC23B (left) and quantification analysis (right) of 40 µg LCLs lysates from probands II.1, families 1 to 8 and four healthy, unrelated controls in six independent experiments accounting for six Western blots performed with independently generated lysates from the one cell line. GAPDH was used as loading control. (**B**) *SEC23B* mRNA quantification in LCLs from proband 2-II.4 is expressed relative to the expression of four healthy, unrelated donors in five independent experiments. *SEC23B* expression levels were normalized to the housekeeping gene *GAPDH*. (**C**) Schematic representation of the effect of the c.1512-3delinsTT and c.1512-16_1512-7delACTCTGGAAT variants (top), which result in the skipping of exons 13 and 14 and a shorter protein product. RT-PCR followed by Sanger sequencing from proband II.1 of family 3 (bottom). (**D**) RT-PCR followed by Sanger sequencing of probands II.1, families 5 and 7. MWM: molecular weight marker. ** *p* < 0.01,**** *p* < 0.0001.

To evaluate the impact of variants on both the *SEC23B* gene and its protein expression, we generated lymphoblastoid cell lines (LCLs) of each patient and four healthy control individuals. Protein and mRNA were analyzed by Western blot and reverse transcription followed by qPCR, respectively. SEC23B protein levels were significantly decreased in the LCLs of all the CDA II patients compared with healthy controls (Figure 4A). Of note, in LCLs of probands II.1 from family 5 and 7 (bearing the nonsense p.Arg535Ter and the frameshift p.Asp693GlyfsTer2 variants, respectively), *SEC23B* mRNA expression levels were significantly decreased with respect to controls (Figure 4B). LCLs from probands II.1 from families 1, 2, 3, and 4 showed no significant change at mRNA level, whereas LCLs from proband II.1, family 8 (carrying the p.Glu109Lys and p.Arg 701H variants) showed significantly increased *SEC23B* mRNA levels compared to controls (Figure 4B).

The proband II.1 from family 3 harbors, in the same allele, the complex intronic variant c.1512-3delinsTT near the intronic deletion c.1512-16_1512-7delACTCTGGAAT inherited from the father; both variants are located in intron 13 of the *SEC23B* gene. An RT-PCR was used to amplify exons 9 to 17 out of *SEC23B* cDNA isolated from patients’ LCLs; this was followed by Sanger sequencing. Results for the mutated *SEC23B* allele indicate skipping of exons 13 and 14 (Figure 4C). Analysis of the RNA sequence by using ExPASy portal [32] confirmed that the mutant mRNA translates into a 681-residue protein isoform, 86 amino acids shorter than the wild type protein.

Only one heterozygous variant was identified in proband II.1 from family 5 (nonsense mutation c.1603C>T (p.Arg535Ter)) and proband II.1 from family 7 (frameshift variant c.2074_2077dupGATG (p.Asp693GlyfsTer2)). In these same patients, *SEC23B* mRNA expression was decreased to approximately half compared to controls (Figure 3B). To investigate if these variants affect the mRNA stability of their mutated alleles, we resourced to RT-PCR to amplify the *SEC23B* cDNA region flanking the c.1603 position for proband II.1, family 5 and the cDNA region c.2074_2077 for proband II.1, family 7 in their respective LCLs samples. As shown in Figure 4D, we were not able to detect the mutated allele in the cDNA of proband II.1 from family 5, suggesting that this allele (carrying the nonsense c.1603C>T (p.Arg535Ter mutation)) might be degraded by the nonsense-mediated decay (NMD) surveillance mechanism. This would account for the halved *SEC23B* mRNA levels observed in Figure 3B. Conversely, RT-PCR done on cDNA samples of proband II.1, family 7 (Figure 4D) detected both the mutated and the wild type allele, hinting that the mutated allele is only partially degraded by NMD but the aberrant protein could be unstable and/or non-functional.

Studies of SEC23B protein levels in CDA II patients with different mutation types (including missense ones) have been previously published [18,26]: in most of these patients, a reduction of SEC23B protein levels was observed. In accordance with these reports, we also observed a reduced amount of SEC23B protein levels in our patients, not always correlated with a reduction at the mRNA level nor compensated by an increase in SEC23A protein production. As different variants may trigger different mechanisms by which the protein is reduced, we postulate that in patients with reduced mRNA levels, reduced protein levels are a consequence of this altered state. In the remaining patients whose mRNA levels are unchanged, the reduced amount of protein may be related to protein instability and/or faster degradation.

Altogether, in the patients we describe here, SEC23B proteins are either more unstable or less effectively translated than in control individuals, accounting for the decreased protein levels and ultimately leading to the disease.

### 2.5. Expression Analysis of SEC23A Paralog in Patients’ LCLs

**Figure 5:**
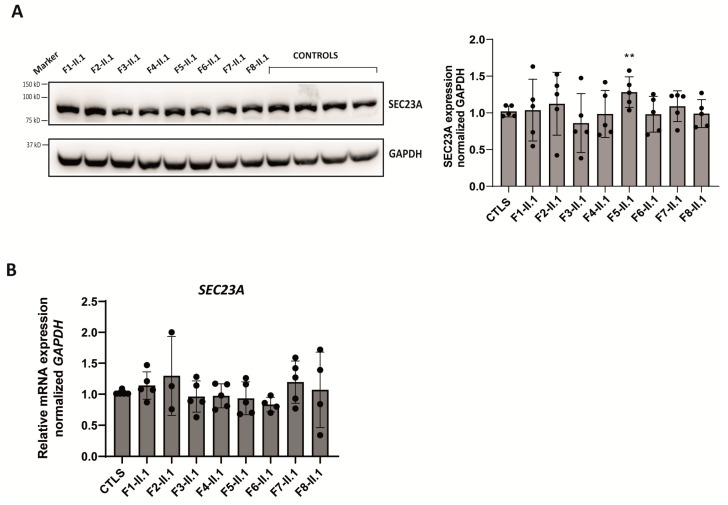
Expression of *SEC23A* mRNA and SEC23A protein. (**A**) Representative Western blot of SEC23A (left) and quantification analysis (right) of 40 µg LCLs lysates from probands II.1, family 1 to 8 and four healthy, unrelated controls in six independent experiments accounting for six Western blots with independently generated lysates from the one cell line. GAPDH was used as loading control. (**B**) *SEC23A* mRNA quantification in LCLs from probands II.1, families 1 to 8 are expressed relative to the expression of four healthy, unrelated donors in five independent experiments performed with independently generated lysates from the one cell line. *SEC23A* expression levels were normalized to the housekeeping gene *GAPDH*. ** *p* < 0.01.

In 2013, Iolascon’s group proposed a SEC23A-mediated compensatory mechanism, which counterbalances SEC23B reduced expression in patients lowly expressing this paralog [26]. This observation was recently replicated in T cells derived from CDA II patients [33]. Given the significantly reduced levels of SEC23B protein found in our patients, the expression of SEC23A was also evaluated in their LCLs. As shown in Figure 5A, a significantly higher expression of SEC23A protein was only observed in LCLs derived from patient 5, despite no change in his mRNA levels (Figure 5B). Every other patient-derived LCLs displayed similar levels of SEC23A protein and mRNA expression to healthy controls (Figure 5).

## 3. Discussion

In this work, we report nine new families including nine probands affected by CDA II and six previously undescribed variants associated with this disease. Out of these novel variants, three are missense (p.Thr445Arg, p.Tyr579Cys, and p.Arg701His), one frameshift (p.Asp693GlyfsTer2), and two affect splicing (c.1512-2A>G and the complex intronic variants c.1512-3delinsTT together with the c.1512-16_1512-7delACTCTGGAAT that are closely located in the same allele). As assessed by computational modeling, the three nonsynonymous substitutions induce protein structure alterations, which range from pronounced in the p.Thr445Arg and p.Arg701His variants to mild in p.Tyr579Cys; all changes potentially impact protein functionality. Out of all the genetic alterations here described, missense variants are the most common (68%), followed by splicing variants (18.75%), and the remaining 12% are either nonsense or frameshift variants. Their frequency spectrum highly correlates with the total frequency of *SEC23B* mutations [34]. The most frequently reported variant in this study is p.Glu109Lys, found in four probands out of nine, three times in a hetero- and once in a homozygous state.

To explore the biological consequences of these newly reported SEC23B variants, we performed protein and mRNA studies in patients’ LCLs. SEC23B protein level was significantly decreased in all the patients’ samples, whereas the corresponding mRNA expression was highly reduced in two patients only (probands II.1, families 5 and 7), both of whom harbor a premature stop codon variant (p.Arg535Ter and p.Asp693GlyfsTer2). Interestingly, only one heterozygous variant in SEC23B was detected in these same probands. This finding deviates from the expected pattern of an autosomal recessive disease, yet other studies have reported analogous CDA II patients [20]. Even so, a second, causative variant cannot be ruled out, this being a deep intronic, a variant in the promoter or an epigenetic change. We showed that the mutated allele of proband II.1, family 5 is fully degraded, accounting for halved mRNA levels and less translated protein; this piece of evidence corroborates the severity of a loss-of-function change. Indeed, nonsense and frameshift mutations in the *SEC23B* gene are much less frequently reported than missense mutations (15%, 14% vs. 65%, respectively) in CDA II patients. Moreover, no patient with two nonsense mutations has ever been reported as total loss of SEC23B expression is expected to harbor a lethal phenotype.

The mammalian SEC23 paralogous proteins (SEC23A and SEC23B) have both closely related and functionally divergent functions. SEC23B-deficient mice pups are born with no obvious anemia and die shortly after birth [35], whereas SEC23A-deficient animals die during mid-embryogenesis with defects in neural tube closure and extraembryonic membrane formation [36]. This discrepancy would account for the different phenotypes observed in response to human mutations. Whereas mutations in the *SEC23B* gene are responsible for CDA II [1], mutations in *SEC23A* result in cranio-lenticulo-sutural dysplasia, a disease characterized by bone abnormalities, in absence of anemia [37]. The *SEC23* genes also share overlapping functions. Studies argue that the disparate phenotypes observed upon their loss are the result of evolutionary shifts in gene-expression programs, rather than unique functions of the SEC23 paralogs [38]. As introduced earlier, a compensatory mechanism between these two duplicate genes has been described. Supported by literature, we set to corroborate functional compensation of SEC23B deficit by SEC23A expression. We measured no significant variation in SEC23A in response to deleterious variants impacting its SEC23B paralog, except for one patient. Although the transcriptional bases of genetic buffering of the *SEC23* genes are not fully elucidated, we consider that mild SEC23B-mutated phenotypes may not be enough to trigger SEC23A compensation. As for our LCLs model system, the two duplicated genes displayed limited functional compensation and ineffective distributed robustness, an outcome possibly owing to the nature of the harbored variants and differences across experimental systems.

This study has some limitations that need to be considered when interpreting the results. No biological material is available from the parents of families 1, 2, 4, and 8. All the patients from these families, here described as compound heterozygotes, present at least one previously described mutation not reported in cis with any other pathogenic or likely pathogenic mutations. The clinical findings present in these probands, together with the pathogenicity of the found variants, suggest that these variants are in trans and are causative of the disease. Concerning the probands from families 5 and 7, the temporal gap existing between the diagnosis done at a young age and the genetic evaluation performed later on in life poses a further limitation to this study as, for these patients with one identified variant, previous reporting of patients with one variant is not full evidence for pathogenicity of the heterozygous status.

Next-generation sequencing (NGS) technology revolutionized the way genetic diagnosis of rare disease as CDAII is done, driving a change from laborious Sanger sequencing to multiplex targeted NGS panels [39]. High-throughput testing options improved the detection of rare pathogenic variants [39,40] and advanced the route to diagnosis, which remains challenged by non-specific symptoms and overlapping clinical phenotypes [41]. With reference to this, diagnosis of all nine patients in this study (some of whom previously misdiagnosed), was achieved by targeted NGS panels following international recommendations [39].

New therapeutic avenues have been explored for the treatment of hereditary anemias. Erythroid maturation agents ameliorate anemia by promoting late-stage erythroid maturation: this mechanism of action makes them amenable to treat beta thalassemia and other myelodysplastic diseases [42]. Novel transforming growth factor (TGF)-β ligand traps, i.e., Luspatercept and Sotatercept are being evaluated as potential CDAII treatments with encouraging results. Luspatercept (Reblozyl^®^) is a recombinant fusion protein that first received FDA approval in 2019 for the treatment of anemia in transfusion-dependent, beta thalassemia patients. A murine analog of Sotatercept, RAP-011, is a TGF-β superfamily inhibitor currently being tested in CDAII cellular models [43].

Novel applications of viral vectors have been encouragingly utilized for therapeutic gene addition/editing in the treatment of red blood cell diseases such as beta thalassemia and pyruvate kinase deficiency (PKD) [44,45]. Innovative therapies such as autologous HSCT of genetically corrected cells are also being explored as an effective treatment for CDA II. Recent work showed that SEC23B lentiviral vector compensates SEC23B deficiency in both SEC23BKO and CDAII hematopoietic progenitor cells [46], breaking ground for gene therapy of autologous hematopoietic stem and progenitor cell (HSPCs) as a feasible, resolutive treatment for CDA II.

In this work, we recapitulate the variants in the *SEC23B* gene causing CDAII. We describe nine new CDA II cases, accounting for six previously undescribed variants, which we characterize both at the functional and computational level. Our findings expand the spectrum of variants amenable to be targeted by therapeutic agents and stem cell-based innovative therapies in CDAII management.

## 4. Materials and Methods

### 4.1. Patients

Written informed consent was obtained for all participants in this study. The study was conducted in accordance with the Declaration of Helsinki’s ethical principles and the protocol was approved by the Ethics Committee associated with the Universitat Internacional de Catalunya on day 04/08/2021. Blood from healthy donors was obtained from the Catalan Tissue and Blood Bank (BST) through an agreement.

### 4.2. DNA Sequencing and Analysis

Patients’ DNA was extracted from peripheral blood using the QIAamp DNA Blood Mini Kit (Qiagen, Valencia, CA, USA) and analyzed using a targeted NGS gene panel (Panel #10040v16) in BloodGenetics S.L. (Esplugues de Llobregat, Barcelona, Spain). The panel included the following genes: *CDAN1*, *C150RF41*, *SEC23B*, *KIF23*, *KLF1*, and *GATA1*. The library was prepared using the Custom HaloPlexTM HS Target Enrichment System (Agilent Technologies, Santa Clara, CA, USA) and sequenced on a MiniSeq platform (Illumina, San Diego, CA, USA). Data were analyzed with SureCall software v 4.2.2 (Agilent Technologies, Santa Clara, CA, USA) and VarSome Clinical software v 11.6.1 (Saphetor SA, Lausanne, Switzerland) [27].

PCR validation of the variants was done with 50 ng of genomic DNA. Primer sequences and PCR conditions are available upon request. The resulting amplification products were verified on a 1% agarose gel with ethidium bromide. PCR products were purified with NucleoSpin^®^ Gel and PCR Clean-up Kit (Macherey-Nagel, GmbH & Co KG, Düren, Germany) and sequenced using the conventional Sanger method (Supplementary Figure S1). Sequencing results were analyzed using Chromas 2.6.6 software (Technelysium Pty Ltd., South Brisbane, Australia).

Variants reported in this study have been submitted to ClinVar (http://www.ncbi.nlm.nih.gov/clinvar, accessed on 19 April 2023).

### 4.3. Generation of Lymphoblastoid Cell Lines (LCLs)

Peripheral blood mononuclear cells (PBMCs) were isolated from patients and healthy unrelated controls from EDTA-treated blood by Lymphoprep^TM^ (StemCell Technologies, Vancouver, BC, Canada) density gradient, following manufacturer’s instructions. PBMCs were grown in RPMI-1640 with 15% heat inactivated FBS, 2 mM L-glutamine, 50 µg/mL streptomycin, 100 U/mL penicillin, and 2.5 µg/mL phytohemagglutinin-L (ThermoFisher Scientific, MA, USA) for seven days. EBV-transformed and immortalized Lymphoblastoid Cell Lines (LCLs) from probands and healthy unrelated controls were established as described in https://unclineberger.org/tissueculture/protocols/, accessed on 19 April 2023.

### 4.4. RNA Extraction, Reverse Transcription, and qPCR

Total RNA was extracted from LCLs with TRIzol^TM^ Reagent (ThermoFisher Scientific, Waltham, MA, USA) following manufacturer’s instructions. First, 1 mg of total RNA was reverse transcribed using GoScript^TM^ Reverse Transcriptase (Promega, Madison, WI, USA) with random primers, following manufacturer’s instructions.

Then, 20 ng of cDNA was subjected to real time quantitative PCR in the CFX96 Real-Time System C1000 Touch Thermal Cycles detection system using the SYBR(R) Green Master mix (both from Bio-Rad, Hercules, CA, USA). Quantitative RT-PCR for *SEC23B* and *SEC23A* mRNA expression was performed using the following primer sets: *SEC23B* Fw AGAACGTCCAGACCTACCTC and Rv AATCAACCTGACAAAGTGGGT; *SEC23A* Fw GCCAAACAACTGCAGGAAATG and Rv CTGTTGGAAGGAGGTGGCT; *GAPDH* Fw ATGGGGAAGGTGAAGGTCG, and Rv GGGGTCATTGATGGCAACAATA. For each gene, patients and control samples were analyzed in the same qRT-PCR and run in duplicate in five independent experiments. Relative quantification (RQ) was calculated with the 2^−∆∆Ct^ method using *GAPDH* as a housekeeping gene. *SEC23B* and *SEC23A* mRNA expression was compared to that of a pool of three controls in each independent experiment.

### 4.5. Immunoblotting

LCLs were washed once with PBS and then resuspended in a lysis buffer containing 20 mM Tris-HCl (pH 7.4), 5 mM EDTA (pH 8), 1% NP40, 150 mM NaCl, and protease inhibitors (A32955, ThermoFisher Scientific, Waltham, MA, USA). Samples were sonicated, followed by a 5-min centrifugation at 4 °C; protein concentration was determined in the supernatant by Bradford assay (Bio-Rad, Hercules, CA, USA). Then, 20 µg total protein lysate was separated by sodium dodecyl sulfate–polyacrylamide gel electrophoresis (SDS-PAGE) (10%) and transferred to polyvinylidene fluoride (PVDF) membranes using the iBlotTM 2 Transfer Device (ThermoFisher Scientific, Waltham, MA, USA). Samples were incubated for 5 min at 95 °C in Laemmli buffer (containing 125 mM Tris-HCl pH 6.8, 10% SDS, 25% glycerol, 1% beta-mercaptoethanol, and bromophenol blue) prior to SDS-PAGE. Transfer of proteins was verified by staining the blots with Ponceau S (Sigma-Aldrich, St. Louis, MO, USA). Immunoblots were incubated with blocking solution (5% skim milk, 0.1% Tween 20, in TBS/TBS-T) for one hour at room temperature before incubation with primary antibodies for 18 h at 4 °C in blocking solution. Primary antibodies included rabbit polyclonal anti–SEC23B (1:1000, ab151258 Abcam, Cambridge, UK), rabbit polyclonal anti–SEC23A (1:5000, ab137583 Abcam Cambridge, UK) and mouse anti–GAPDH (1:30,000, 60004-1-Ig Proteintech, Manchester, UK), this latter used as a loading control. The blots were then washed with TBST and incubated during one hour at room temperature with the appropriate horseradish peroxidase (HRP)-labeled secondary antibody in blocking solution (1:5000, peroxidase conjugated anti-rabbit IgG (H+L) and 1:30,000 peroxidase conjugated anti-mouse IgG (H+L)), both from Jackson ImmunoResearch. Immunodetection was done on Immobilon(R) Forte Western HRP Substrate (Merck Millipore, Burlington, MA, USA), images were taken with ChemiDoc^TM^ and quantified using the software Image Lab v 5.2 (both from Bio-Rad, Hercules, CA, USA). Six independent experiments were carried out, in which all the eight patients and four control samples were included. The same controls were used across the experiments. Patients’ SEC23B and SEC23A expression was calculated relative to the mean of the four controls for each experiment.

### 4.6. Computational Studies

The tridimensional structure of the human SEC23B protein was obtained from the AlphaFold Protein Structure Database (https://alphafold.ebi.ac.uk/, accessed on 19 April 2023) [47]. The p.Thr445Arg, p.Tyr576Cys, and Arg701His variants were introduced in the SEC23B model using the PyMol v 2.5 software. The resulting mutated structures were refined using the PyMol algorithms and the H-bonds of both wild-type and mutated residues were calculated.

### 4.7. Statistics

GraphPad Prism 9.0.0.121 software (GraphPad Software) was used for statistical analysis. All data were reported as mean with standard error of the mean or stated otherwise. The specific statistical analysis used is stated in each figure’s legend. *p*-values were represented as: ns > 0.05, * < 0.05, ** < 0.01, *** < 0.001, **** < 0.0001.

## Figures and Tables

**Table 1:** Clinical data and pathogenic variants of patients diagnosed with CDA II.

Prob.	S	Age (Years)	Hb (g/L)	MCV (fL)	RBC (10^6^/μL)	Total Bilirubin (mg/dL)	Dir. Bilirubin (mg/dL)	LDH (UI/dL)	Haptoglobin (mg/dL)	Serum Iron (μg/dL)	Serum Ft (μg/L)	Fetal Hb	Nucleotide Change/Protein
F1-II.1	F	(a) 15	(a) N.A.	(a) N/A	(a) N/A	(a) N/A	(a) N/A	(a) N/A	(a) N/A	(a) N/A	(a) N/A	N/A	c.325G>A(p.Glu109Lys); c.1334C>G(p.Thr445Arg)
		(b) 25	(b) 90	(b) 96.1	(b) 3.06	(b) 3.16	(b) 0.62	(b) 242	(b) <0.3	(b) 256	(b) 647	N/A	
F2-II.1	M	(a) 0	(a) 85	(a) 85	(a) N/A	(a) N/A	(a) 0.46	(a) 431.2	(a) N/A	(a) 64	(a) 525	(d) 1%	c.1736A>G(p.Tyr579Cys); c.835-2A>G
		(b)12	(b) N/A	(b) N/A	(b) 3.11	(b) 4.26	(b) N/A	(b) N/A	(b) N/A	(b) N/A	(b) N/A	N/A	
F3-II.1	M	(a) 32	(a) 130	(a) 91	(a) 4.29	(a) 3.03	(a) 0.51	(a) 202	(a) <6	(a) 156	(a) 932	N/A	c.325G>A(p.Glu109Lys); c.1512-3delinsTT + c.1512-16_1512-7delACTCTGGAAT
		(b) 42	(b) 127	(b) 90	(b) 4.15	(b) 2.656	(b) N/A	(b) 1.04	(b) <1	(b) 144	(c) 297	N/A	
F4-II.1	F	(a) 25	(a) 82	(a) 90	(a) 3.50	(a) 2.92	(a) 0.87	(a) N/A	(a) 0.00	(a) 156.36	(a)	N/A	c.40C>T(p.Arg14Trp); c.1968T>G(p.Phe656Leu)
		(b) 60	(b) 122	(b) 90.1	(b) 3.85	(b) 1.07	(b) N/A	(b) N/A	(b) N/A	(b) 183.17	(b) 51	N/A	
F5-II.1	M	(a) 21	(a) N/A	(a) N/A	(a) N/A	(a) N/A	(a) N/A	(a) N/A	(a) N/A	(a) N/A	(a) N/A	N/A	c.1603C>T(p.Arg535Ter)
		(b) 52	(b) 127	(b) 90.6	(b) 4.20	(b) 5.9	(b) 1.2	(b) 275	(b) <8	(b) 158	(b) 40	N/A	
F6-II.1	F	(a) 21	(a) 110	(a)103	(a) 3.46	(a) 3.34	(a) 0.67	(a) 129	(a) 23	(a) 217	(a) 242	N/A	c.325G>A(p.Glu109Lys); c.325G>A(p.Glu109Lys)
		(b) 30	(b) 113	(b) 104	(b) 3.45	(b) 3.18	(b) 1.12	(b) 151	(b) N/A	(b) 196	(b) 153	N/A	
F7-II.1	M	(a) 10	(a) 833	(a) 91	(a) 2.87	(a) 2.4	(a) 1.3	(a) 390	(a) 38	(a) 105	(a) 178	N/A	c.2074_2077dupGATG(p.Asp693GlyfsTer2)
		(b) 53	(b) 105	(b) 98.5	(b) 3.34	(b) 3.9	(b) 0.9	(b) 176	(b) N/A	(b) 227	(b) 469	N/A	
F8-II.1	F	(a) 8	(a) 95	(a) 95	(a) 2.92	(a) 4.99	(a) 0.59	(a) < 500	(a) 10	(a) 67	(a) 74	(a) 0.5%	c.325G>A(p.Glu109Lys); c.2102G>A (p.Arg701His)
		(b) 24	(b) 78	(b) 106	(b) 2.13	(b) 4.82	(b) 0.45	(b)167	(b)	(b) 69	(b) 659	N/A	
F9-II.1	M	(a) 9	(a) 106	(a) 83	(a) 3.45	(a) 2.0	(a) 0.5	(a) 430	(a) 15.2	(a) 241	(a) 37.4	(a) 1.3	c.40C>T(p.Arg14Trp); c.1512-2A>G
		(b) 24	(b) 99	(b) 93.8	(b) 3.29	(b) 4.3	(b) 0.4	(b) 167	(b) 56	(b) 228	(b) 142	(b) N/A	

Prob: proband. S: sex (M male, F female). (a): at diagnosis, (b): last visit. Hb: hemoglobin. MCV: mean corpuscular volume. RBC: red blood cells. Dir: direct. LDH: lactate deshidrogenase. Ft: ferritin. N/A: not available.

## Data Availability

The data presented in this study are available on request from the corresponding author.

## Supplementary Materials

The following supporting information can be downloaded at: https://www.mdpi.com/article/10.3390/ijms24129935/s1.
