# Supplementary material for: New Cases and Mutations in SEC23B Gene Causing Congenital Dyserythropoietic Anemia Type II
Source: Int J Mol Sci. 2023 Jun 9;24(12):9935. doi: 10.3390/ijms24129935 (PMC10298408; doi:10.3390/ijms24129935)
Supplement: Supplementary file 1 [file ijms-24-09935-s001.zip › ijms-2384944-supplementary.pdf]

Supplementary data

New Cases and Mutations in *SEC23B* Gene Causing Congenital Dyserythropoietic Anemia Type II

Melina Mara Musri [1,†,‡], Veronica Venturi [2,†], Xènia Ferrer-Cortès [1,2], Lídia Romero-Cortadellas [2], Gonzalo Hernández [1,2], Pilar Leoz [3], María Pilar Ricard Andrés [4], Marta Morado [5], María del Carmen Fernández Valle [6], David Beneitez Pastor [7], Ana Ortuño Cabrero [7], Maite Moreno Gamiz [8], Leonor Senent Peris [9], Amanda Isabel Perez-Valencia [10], Santiago Pérez-Montero [1], Cristian Tornador [1] and Mayka Sánchez [1,2,*]

Supplementary Figure 1. Validation of patients' mutations by Sanger sequencing.

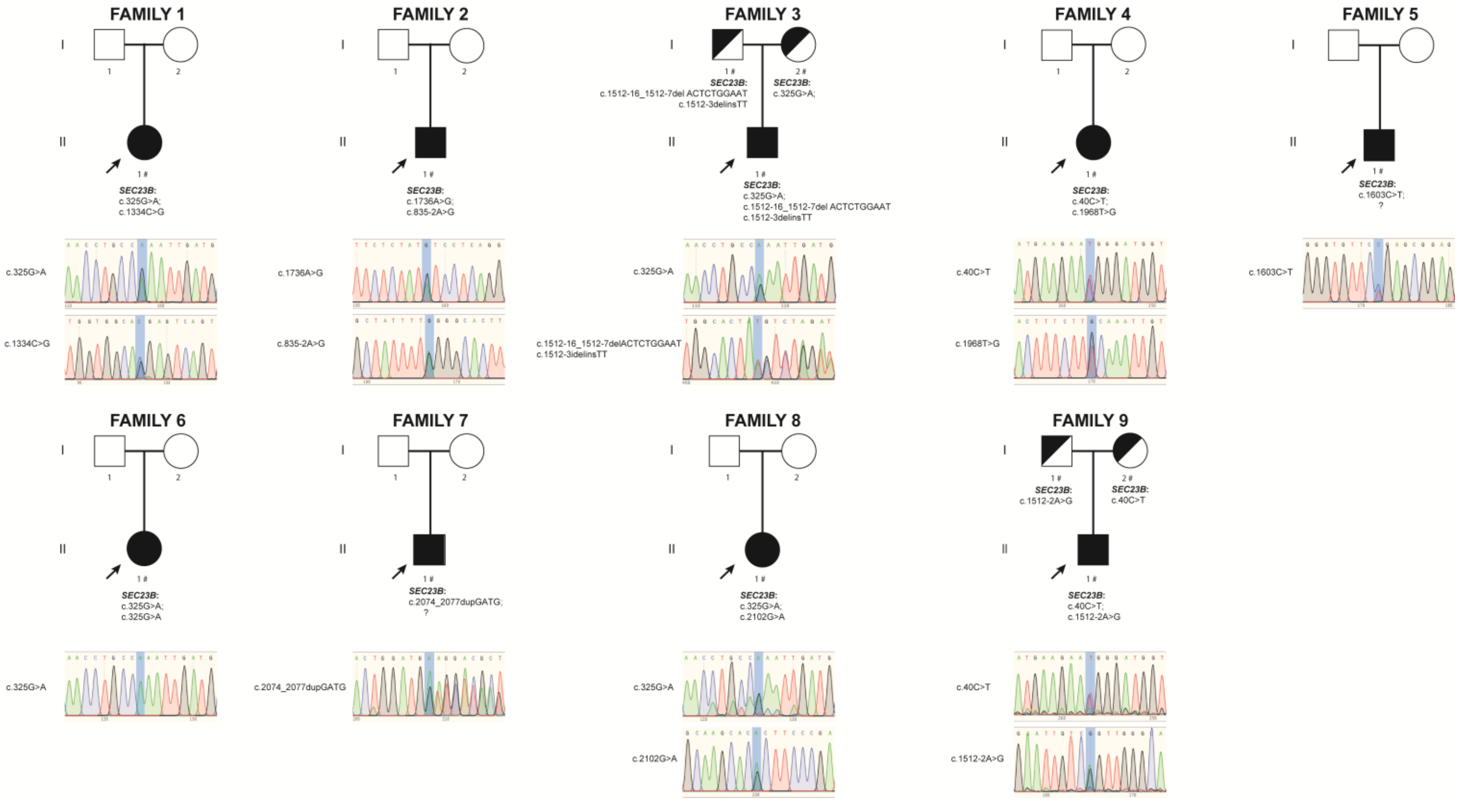

Supplementary Table 1. Reported mutations in *SEC23B*.

Exon	Nucleotide change	AA change	Domain	Reference
**Missense mutations**				
2	c.1A>G	p.Met1Val	Segment 1	Bianchi et al 2016
2	c.40C>T	p.Arg14Trp	Segment 1	Schwarz et al 2009
2	c.52C>T	p.Arg18His	Segment 1	Schwarz et al 2009
2	c.53G>A	p.Arg18Cys	Segment 1	Bianchi et al 2016
2	c.74C>A	p.Pro25His	Segment 1	Liu et al 2012
2	c.122T>C	p.Leu41Pro	Segment 1	Bianchi et al 2016
2	c.197G>A	p.Cys66Tyr	Zinc finger	Fermo et al 2010
2	c.211A>C	p.Asn71His	Zinc finger	Bianchi et al 2016
3	c.221G>A	p.Cys74Tyr	Zinc finger	Russo et al 2013
3	c.247T>G	p.Trp83Gly	Zinc finger	Bianchi et al 2016
4	c.325G>A	p.Glu109Lys	Segment 2	Schwarz et al 2009
5	c.490G>T	p.Val164Leu	Trunk	Moreno Carralero et al 2018
5	c.494G>A	p.Gly165Asp	Trunk	Wang et al 2018
5	c.569G>A	p.Arg190Gln	Trunk	Moreno Carralero et al 2018
7	c.716A>G	p.Asp239Gly	Trunk	Schwarz et al 2009
8	c.887C>T	p.Pro296Leu	Trunk	Bianchi et al 2016
8	c.938G>A	p.Arg313His	Trunk	Schwarz et al 2009
8	c.953T C	p.Ille318Thr	Trunk	Schwarz et al 2009
9	c.1043A>C	p.Asp348Ala	Trunk	Bianchi et al 2009
9	c.1109G>C	p.Gly370Ala	Trunk	Bianchi et al 2016
10	c.1157A>G	p.Gln386Arg	Trunk	Schwarz et al 2009
10	c.1162T>A	p.Phe388Ile	Trunk	Chen et al 2021
11	c.1254T>G	p.Ille418Met	Beta sheet	Russo et al 2010
11	c.1307C>T	p.Ser436Leu	Beta sheet	Russo et al 2010
**11**	**c.1334C>G**	**p.Thr445Arg**	**Beta sheet**	**This study**
11	c.1352G>T	p.Cys451Phe	Beta sheet	Wang et al 2018
12	c.1385A>G	p.Tyr462Cys	Beta sheet	Schwarz et al 2009
13	c.1415C>T	p.Pro472Leu	Beta sheet	Russo et al 2014
12	c.1445A>G	p.Gln482Arg	Beta sheet	Wang et al 2018
13	c.1453A>G	p.Thr485Ala	Beta sheet	Punzo et al 2011
13	c.1467C>G	p.His489Gln	Beta sheet	Wang et al 2018
13	c.1489C>T	p.Arg497Cys	Beta sheet	Schwarz et al 2009
13	c.1508G>A	p.Arg503Gln	Beta sheet	Iolascon et al 2010
14	c.1571C>T	p.Ala524Val	Helical	Schwarz et al 2009
14	c.1588C>T	p.Arg530Gln	Helical	Iolascon et al 2010

14	c.1589G>A	p.Arg530Trp	Helical	Schwarz et al 2009
14	c.1636C>T	p.Arg546Trp	Helical	Moreno Carralero et al 2018
14	c.1654C>T	p.Leu552Phe	Helical	Russo et al 2010
15	c.1676T>C	p.Phe559Ser	Helical	Bianchi et al 2016
15	c.1685A>G	p.Tyr562Cys	Helical	Iolascon et al 2010
15	c.1727T>C	p.Phe576Ser	Helical	Wang et al 2018
15	c.1733T>C	p.Leu578Pro	Helical	Russo et al 2010
15	c.1735T>A	p.Leu579Asn	Helical	Russo et al 2010
**15**	**c.1736A>G**	**p.Tyr579Cys**	**Helical**	**This study**
16	c.1754A>G	p.His585Arg	Helical	Bianchi et al 2016
16	c.1808C>T	p.Ser603Leu	Helical	Bianchi et al 2009
16	c.1831C>T	p.Arg611Trp	Helical	Li et al 2017
16	c.1832G>C	p.Arg611Pro	Helical	Russo et al 2010
16	c.1832G>A	p.Arg611Gln	Helical	Moreno Carralero et al 2018
16	c.1858A>G	p.Met620Val	Helical	Russo et al 2010
16	c.1859T>C	p.Met620Thr	Helical	Zhang et al 2018
17	c.1910T>G	p.Val637Gly	Gelsolin	Punzo et al 2011
17	c.1949T>C	p.Leu650Ser	Gelsolin	Wang et al 2018
17	c.1964T>C	p.Phe655Ser	Gelsolin	Bianchi et al 2016
17	c.1968T>G	p.Phe656Leu	Gelsolin	Iolascon et al 2010
18	c.2005 T>C	p.Trp669Arg	Gelsolin	Russo et al 2014
18	c.2101C>T	p.Arg701Cys	Gelsolin	Bianchi et al 2009
**18**	**c.2102G>A**	**p.Arg701His**	**Gelsolin**	**This study**
17	c.2108C>T	p.Pro703Leu	Gelsolin	Wang et al 2018
18	c.2129C>T	p.Thr710Met	Gelsolin	Amir et al 2011
19	c.2166A>C	p.Lys723Gln	Segment 6	Iolascon et al 2010
19	c.2180C>T	p.Ser727Phe	Segment 6	Punzo et al 2011
20	c.2270A>C	p.His757Pro	Segment 6	Russo et al 2010
**Nonsense mutations**				
2	c.28C>T	p.Gln10Ter	Segment 1	Bianchi et al 2016
2	c.71G>A	p.Trp24Ter	Segment 1	Liu et al 2012
3	c.235C>T	p.Arg79Ter	Zinc finger	Schwarz et al 2009
5	c.367C>T	p.Arg123Ter	Zinc finger	Bianchi et al 2016
5	c.568C>T	p.Arg190Ter	Trunk	Bianchi et al 2009
6	c.640C>T	p.Gln214Ter	Trunk	Punzo et al 2011
6	c.649C>T	p.Arg217Ter	Trunk	Schwarz et al 2009
7	c.790C>T	p.Arg264Ter	Trunk	Schwarz et al 2009
8	c.923T>G	p.Leu308Ter	Trunk	Bianchi et al 2016
8	c.970C>T	p.Arg324Ter	Trunk	Schwarz et al 2009
9	c.1015C>T	p.Arg339Ter	Trunk	Russo et al 2010
10	c.1201C>T	p.Arg401Ter	Segment 3	Schwarz et al 2009
13	c.1428_1429ins22	p.Gly477Ter	Beta sheet	Bianchi et al 2016

14	c.1561C>T	p.Gln521Ter	Helical	Moreno Carralero et al 2018
14	c.1603C>T	p.Arg535Ter	Helical	Russo et al 2010
14	c.1648C>T	p.Arg550Ter	Helical	Iolascon et al 2010
14	c.1660C < T	p.Arg554Ter	Helical	Bianchi et al 2009
19	c.2152C>T	p.Arg718Ter	Gelsolin	Bianchi et al 2009
**Frameshift mutations**				
3	c.222-817_366 + 4242del	p.Gln75GlufsTer7	Zinc finger-Trunk	Schwarz et al 2009
5	c.387delG	p.Leu129LeufsTer26	Trunk	Russo et al 2010
5	c.428delAinsCG	p.Asp143AlafsTer35	Trunk	Bianchi et al 2016
5	c.489delG	p.Val164TrpfsTer3	Trunk	Aydin Koker et al 2018
5	c.536_537insTAAG	p.Cys180LysfsTer3	Trunk	Bianchi et al 2016
9	c.1063delG	p.Asp355IlefsTer8	Trunk	Schwarz et al 2009
10	c.1129_1130del GA	p.Asp377PhefsTer17	Trunk	Bianchi et al 2016
13	c.1419_1423insC	p.Ile473IlefsTer47	Beta sheet	Russo et al 2013
16	c.1821delT	p.His608IlefsTer7	Segment 5	Bianchi et al 2009
17	c.1962-64delT	p.Thr654ThrfsTer13	Gelsolin	Iolascon et al 2010
17	c.1989_1990insT	p.Glu664Ter	Gelsolin	Bianchi et al 2016
**18**	**c.2074_2077dupGATG**	**p.Asp693GlyfsTer2**	**Gelsolin**	**This study**
19	c.2150delC	p.Ala717ValfsTer7	Gelsolin	Russo et al 2010
20	c.2262delC	p.Phe754LeufsTer5	Segment 6	Bianchi et al 2016
20	c.2254_2255insA	p.Val753GlifsTer17	Segment 6	Wang et al 2018
**Indels**				
7	c.733_735del CTT	p.Leu245del	Trunk	Bianchi et al 2016
14	c.1603delC	p.Arg535del	Helical	Chen et al 2021
16	c.1857_1859delCAT	p.Ille619del	Segment 4	Russo et al 2010
20	c.2243_2245delATG	p.Asp748ValVal749del	Segment 6	Bianchi et al 2016
**Splicing mutations**				
Intron 2	c.221 + 31A>G	New donor site creation (->0.99)*		Russo et al 2010
Intron 2	c.221+163A>G	Cryptic donor site (1.79>8.95) Δ +400 #		Russo et al 2013
Intron 2	c.222-78C>T	RNA Decay*		Russo et al 2014
Intron 3	c.279 + 3A>G	Donor site alteration (1.00>0.95)e		Russo et al 2010
Intron 3	c.279+5 G>A	aberrant splicing		Bianchi et al 2016
Intron 4	c.367-1G>A	aberrant splicing		Bianchi et al 2016
Intron 6	c.689 + 1G>A			Schwarz et al 2009
Intron 6	c.689 + 1G>C	disease causing		Yehia et al 2015
Intron 7	c.834+3A>C	Donor splice site (8.88>3.31) – Δ 62.7#		Russo et al 2013

Intron 7	c.835-2A>G	Aberrant splicing	Bianchi et al 2016
Intron 9	c.1109+1G>A	Donor site abolition (0.93>–)e	Russo et al 2010
Intron 9	c.1109+5G>A	Donor site abolition (0.93>–)e	Russo et al 2010
Intron 13	**c.1512-2A>G**		**This study**
Intron 13	**c.1512-3delinsTT**		**This study**
Intron 18	c.2149-2A>G		Zhang et al 2018
**Regulatory**			
5′UTR	c.-589A>G		Russo et al 2014
5′UTR	c.-34G>A		Russo et al 2014

* Splice Site Prediction by Neural Network (WT sequence score > mutated sequence score).
#HSF, Human Splicing Finder (http://www.umd.be/HSF/).
